# More Evidence for the Standard Account of Theory of Mind Development: A Reply to Fabricius (2026)

**DOI:** 10.1111/cogs.70270

**Published:** 2026-10-07

**Authors:** Hannes Rakoczy, Lydia Schidelko

**Affiliations:** ^1^ Institute of Psychology University of Göttingen

**Keywords:** Theory of Mind, Psychology, Cognitive psychology, Cognitive development

## Abstract

Fabricius argues that the results of a recent study of ours speak for a deflationary competence limitation account of Theory of Mind development. According to this account, children do not operate with proper mental state concepts until age 6–7 or older. However, the original findings are, in fact, incompatible with such competence limitation accounts, but compatible with the standard picture of Theory of Mind development: According to this picture, children acquire proper mental state concepts by age 4, but this competence is sometimes masked by performance factors.

In his commentary, Fabricius (2026) argues that the results of a recent study of ours (Schidelko & Rakoczy, 2025) speak for his “perceptual access reasoning” account. According to this deflationary competence limitation account, children do not have the competence to operate with proper mental state concepts until age 6–7 or older. We disagree. Here, we take the opportunity to explain in which ways the findings are, in fact, incompatible with deflationary accounts, but compatible with the standard picture of Theory of Mind development: According to this picture, children acquire proper mental state concepts by age 4, but this competence can be masked by performance factors.

## 1 What is the theoretical background debate?

The theoretical debate in the background starts from the following explanandum, a puzzling set of empirical findings: once children come to master what is considered the litmus test of Theory of Mind, the false belief test, they begin to fail trivial control true belief tasks (e.g., Fabricius, Boyer, Weimer, & Carroll, 2010; Oktay‐Gür & Rakoczy, 2017). Why is that?

The standard account is a performance limitation account: Children this age have acquired the metarepresentational competence to ascribe mental states, as indicated in their mastery of the false belief test and many related tasks (Perner, 1991; Rakoczy, 2022). Their failure in true belief tasks reflects some kind of performance limitations, most plausibly to do with task pragmatics such as trivial, confusing, academic test questions (Oktay‐Gür & Rakoczy, 2017; Rakoczy, 2017; Rakoczy & Oktay‐Gür, 2020). Fabricius, in contrast, argues for a deflationary competence limitation account: children before age 6–7 do not yet have a proper Theory of Mind competence and no mental state concepts. All they have is simpler heuristics: first, before age 4, only “reality‐based reasoning” (agents will act in accordance with reality, e.g., search for desired objects where they are), and then, from age 4 to 6, also “perceptual access reasoning” (if agents have full perceptual access to a scene, they will act successfully; if not, they will act unsuccessfully). Children age 4–6 thus may look like they apply proper mental state concepts; but, in reality, they are only using the simple perceptual access heuristics which allows them to solve false belief tasks (the agent has not had perceptual access…and thus makes a mistake); crucially, however, the same heuristics will make them now fail many true belief tasks—those in which an agent has a true belief but has not had uninterrupted perceptual access (the agent has not had perceptual access…and thus makes a mistake).

## 2 **What do the main analyses in** Schidelko and Rakoczy (2025) **show?**

Previous evidence mostly came from local studies on individual aspects of true belief tasks and was generally mixed. Some studies found evidence for the standard picture: once the true belief tasks were administered nonlinguistically, for example, and thus no pragmatic factors were in play, children did not show any difficulty with the tasks (Rakoczy & Oktay‐Gür, 2020). When the tasks pragmatics was manipulated in various ways to make the true belief test question less odd and confusing, children's difficulties disappeared (Oktay‐Gür & Rakoczy, 2017; Rakoczy & Oktay‐Gür, 2020). Other studies, in contrast, found evidence in line with the deflationary account: when factors deemed important by that account (e.g., whether or not the agent left at some irrelevant point in the narrative; whether or not this was highlighted by the experimenter) were manipulated, children showed specific difficulties with true belief tasks under conditions predicted by the account (e.g., when the agent left, and this was not highlighted (Fabricius et al., 2010, 2021)).

The rationale of Schidelko and Rakoczy (2025) was thus to test between the accounts in more systematic and stringent ways. To this end, factors deemed relevant by the two accounts were manipulated independently: First, according to the pragmatic performance limitation account, it makes a crucial difference whether the true belief question makes pragmatic sense or is pragmatically confusing. One way of making it meaningful and less confusing is by embedding it in a more complicated scenario in which there are two agents, one with a true belief, and the other one with a false belief. In this way, due to the contrast, asking about the one agent's true belief makes much more sense pragmatically. Though more complex, this condition should be easier then. The first factor was thus manipulated such that there were baseline conditions in which there were pragmatic performance factors since the test question was an academic test question about a trivial true belief (trivial+), and other conditions in which these pragmatic performance factors were removed and the true belief test questions were more meaningful since there were two actors, one with a true, and another with a false belief (trivial−). Second, according to the deflationary perceptual access reasoning account, it should make a difference, in particular, whether the agent leaves and whether this is highlighted by the experimenter. If the agent leaves, and this is not highlighted, this should lead to characteristic true belief errors. The contrast here was thus between conditions (PAR+) in which the agent left, and this was not highlighted by the experimenter (and some other manipulations deemed relevant) and conditions in which this was not the case (PAR−). The two manipulations were crossed in a 2×2 design.

The main analyses focused, in the 2×2 design, on the independent effects highlighted by the pragmatic performance limitation account, and by the perceptual access reasoning account. Both kinds of factors made a difference, as indicated by the two main effects. Only in the baseline condition (trivial+/PAR+), in which the factors deemed difficult by both types of account were realized, did children perform poorly below chance levels. Overall, once one or both sets of factors deemed to make the task easier by the two accounts were modified, children's difficulties were alleviated and they performed above chance. What do these findings mean? Well, they speak for both kinds of account to some degree. But they are not decisive either way.

## 3 What do the exploratory analyses of the most informative condition show?

Crucially, however, in addition to this general analysis that remains indecisive, there was one condition that was more informative and that was the target of additional analyses (unfortunately, at the time of the preregistration, we did not yet make the logic of this condition with its power to decide between the accounts clear enough—which is why the corresponding analyses are, formally speaking, “exploratory”). For this crucial condition, the two accounts make clearly competing predictions: the condition in which the pragmatic performance factors were alleviated, but not the PAR‐factors (trivial−/PAR+). The pragmatic performance limitation account predicts that this condition, even though complex in terms of working memory (one needs to coordinate and remember two story lines about two agents in the narrative), should pose no difficulty for those children who operate with true mental state concepts: they should be able to ascribe beliefs to the agents in principle; so they should ascribe false beliefs to one agent; and through the contrast, ascribing true beliefs to the other agent should now be pragmatically unproblematic. Within a given scenario, they should thus answer both True Belief (TB) and False Belief (FB) questions correctly: TB+/FB+. In contrast, the deflationary perceptual access reasoning account predicts that children necessarily fail this condition for the following reasons: First, they do not have proper mental state concepts to truly solve the tasks. Second, there is no simpler heuristics that would allow them to solve the task in simpler ways. Reality‐based reasoning would give you only half the questions right (TB+/FB−), and perceptual access reasoning equally so, just the other way around (TB−/FB+).

Empirically, the results are clear: children perform proficiently in this condition. They solve both true belief and false belief questions within the same scenario. Theoretically, this is in line with the pragmatic performance limitation account. It is incompatible with the perceptual access reasoning account.

## 4 Can the results of the most informative condition be explained away?

In his commentary, Fabricius (2026) tries to explain this crucial result away in the following way: Perhaps the proficient performance of the children in this condition is merely due to a subpopulation of older children (age 6 and 7) who have already acquired metarepresentational Theory of Mind. The younger children, in contrast, do behave in accordance with the simpler perceptual access reasoning heuristics. If this were the case, one should see crucial differences between younger and older children's performance in this condition: older children who operate with Theory of Mind concepts should solve both FB and TB tasks, whereas younger children who use perceptual access reasoning should show the characteristic pattern: solve FB tasks, fail TB.

In a reanalysis of the data, Fabricius claims to find exactly such a pattern: “…it is not surprising that the mean was above chance, because the age range extended to 7½ years and would include belief reasoners. The publicly available data confirm that only the 7½‐year‐olds (*M* = 0.90) performed as predicted by the pragmatic account. The 4½‐year‐olds (0.55) did not differ from chance” (p. 4).

However, this is not the right kind of reanalysis that is needed to address the question under discussion (are there age differences in the proportions of children who solve both FB and TB tasks?). The reason this is the wrong analytical approach is the following: This reanalysis uses the whole samples tested at the corresponding ages irrespective of children's performance in the false belief screening test. That means that both those children who failed and those who passed the FB screening task are lumped together. In this way, however, the crucial point of the analysis (“once children solve FB tasks, do they fail or pass TB tasks?”) gets lost. The appropriate analyses, in contrast, should be run only on the subsample of children who did pass the false belief screening. This is the subpopulation of theoretical interest for the target question: why children, once they master false belief tasks, fail true belief tasks.

A closer look at the different age groups’ performance in the crucial (trivial−/PAR+) condition in relation to their performance in the FB screening task reveals the following: Naturally, the proportion of children who failed the false belief screening task are higher in younger age groups. So, yes, the whole population of 4‐year‐olds performed less proficiently in true belief tasks than the whole population of 7‐year‐olds. But among the 4‐year‐olds, there was a considerable subpopulation who had failed false belief screening tasks, whereas among the 7‐year‐olds, there were hardly any. If one then focuses, as one should, and as we did for the focal analyses in the original paper, only on the subpopulations of those children who passed false belief screening tasks, the results are, again, quite clear: there were no such age effects in true belief task performance; all age groups performed proficiently (≥75% correct). The proper reanalysis thus corroborates the original interpretation of the findings as evidence for the pragmatic performance limitation account.

## 5 Conclusion

All in all, the present results are thus in part compatible with both types of accounts. The two main effects of the main analysis indicate that both factors deemed relevant by the pragmatic performance limitation account and those emphasized by Fabricius’ deflationary competence limitation account make a difference to children's performance in true belief tasks. However, in another crucial respect, they are incompatible with competence limitation accounts but compatible with pragmatic performance limitation accounts: for one condition (trivial−/PAR+), the pragmatic performance limitation predicts the successful performance that was observed, which the competence limitation account cannot explain. An attempt by Fabricius to explain away the results of this crucial condition is based on false premises and thus invalid. Once properly analyzed, the findings from this condition are corroborated and present more evidence for the standard picture of Theory of Mind development: children from around age 4 acquire metarepresentational Theory of Mind competence, but this competence can be masked by pragmatic performance factors.

## 6 Outlook

In broader context, however, the present findings may be compatible both with the standard picture of Theory of Mind development, and with a modified version of the perceptual access reasoning account. Such a modified version would not be a competence limitation account. The claim would not be that children before age 6–7 lack, in principle, the metarepresentational capacity to ascribe mental states. Rather, it would be a performance limitation account: even though children have the competence, this gets sometimes masked by their use of simpler heuristics. More systematic future studies are needed to explore the prospects of such a more nuanced and conciliatory picture.
